# Sodium nitroprusside modulates oxidative and nitrosative processes in *Lycopersicum esculentum* L. under drought stress

**DOI:** 10.1007/s00299-024-03238-3

**Published:** 2024-05-28

**Authors:** Cengiz Kaya, Ferhat Uğurlar, Chandra Shekhar Seth

**Affiliations:** 1 Soil Science and Plant Nutrition Department, Harran University, Şanlıurfa, 63200 Turkey; 2 Department of Botany, University of Delhi, New Delhi, Delhi 110007 India

**Keywords:** Nitric oxide, Non-enzymatic antioxidants, ROS, RNS, S-Nitrosylation, Tomato plants

## Abstract

**Key message:**

Sodium nitroprusside mediates drought stress responses in tomatoes by modulating nitrosative and oxidative pathways, highlighting the interplay between nitric oxide, hydrogen sulfide, and antioxidant systems for enhanced drought tolerance.

**Abstract:**

While nitric oxide (NO), a signalling molecule, enhances plant tolerance to abiotic stresses, its precise contribution to improving tomato tolerance to drought stress (DS) through modulating oxide-nitrosative processes is not yet fully understood. We aimed to examine the interaction of NO and nitrosative signaling, revealing how sodium nitroprusside (SNP) could mitigate the effects of DS on tomatoes. DS-seedlings endured 12% polyethylene glycol (PEG) in a 10% nutrient solution (NS) for 2 days, then transitioned to half-strength NS for 10 days alongside control plants. DS reduced total plant dry weight, chlorophyll a and b, *Fv/Fm,* leaf water potential (Ψ_I_), and relative water content, but improved hydrogen peroxide (H_2_O_2_), proline, and NO content. The SNP reduced the DS-induced H_2_O_2_ generation by reducing thiol (–SH) and the carbonyl (–CO) groups. SNP increased not only NO but also the activity of l-cysteine desulfhydrase (L-DES), leading to the generation of H_2_S. Decreases in S-nitrosoglutathione reductase (GSNOR) and NADPH oxidase (NOX) suggest a potential regulatory mechanism in which _S_-nitrosylation [formation of S-nitrosothiol (SNO)] may influence protein function and signaling pathways during DS. Moreover, SNP improved ascorbate (AsA) and glutathione (GSH) and reduced oxidized glutathione (GSSG) levels in tomato plants under drought. Furthermore, the interaction of NO and H_2_S, mediated by L-DES activity, may serve as a vital cross-talk mechanism impacting plant responses to DS. Understanding these signaling interactions is crucial for developing innovative drought-tolerance strategies in crops.

## Introduction

Tomatoes, a cornerstone of global agriculture, face production challenges due to drought stress, which is a critical factor limiting their yield and quality (Ahanger et al. 2021). Understanding the adaptive mechanisms of tomato plants to drought stress is therefore essential. In particular, the role of NO in enhancing drought tolerance presents a promising avenue for research, offering potential strategies to bolster resilience in this vital crop. Drought stress presents a substantial challenge to agriculture, especially in regions marked by limited water sources for irrigation, including arid and semi-arid regions (Samimi et al. 2020). Its potential for adverse impacts extends to plant growth, photosynthetic activity and fruit production, ultimately resulting in economic losses and diminished crop yields (Ahluwalia et al. 2021; Seleiman et al. 2021). The escalating global population and the consequences of global warming, including increasing temperatures and changing precipitation patterns, have contributed to a continuous reduction in the available water for agricultural crop irrigation (Islam and Karim 2019). DS can harm plants by disrupting the delicate balance between reactive oxygen species (ROS) and compensatory antioxidants (Razi and Muneer 2021), potentially leading to physiological imbalances, but antioxidants are involved in modulating ROS levels and safeguarding the plant against oxidative damage (Jahan et al. 2023).

Biostimulators exhibit promising capability to alleviate the adverse consequences of different stressors, especially water stress. They achieve this by fostering plant growth and elevating the enzymes’ activities linked to stress response pathways, ultimately enhancing plant tolerance to drought (Jafari et al. 2022; Choudhary et al. 2024). Nitric oxide (NO) has been specifically focused in this research paper to control and regulate drought and water stress in tomato plants due to its unique role as a biostimulator. NO is a pivotal signaling molecule that not only enhances plant tolerance to abiotic stresses but also actively participates in various stress responses, including drought stress (DS). Its ability to modulate antioxidant enzyme activities helps in mitigating oxidative damage caused by reactive oxygen species (ROS), which is crucial during DS (Rezayian et al. 2020). Furthermore, the interplay between NO and hydrogen sulfide (H_2_S) in regulating plant physiology has been gaining attention, as this interaction influences mutual production and impacts downstream signaling pathways, potentially leading to enhanced stress tolerance (Marcolongo et al. 2019; Sun and Li 2022). Therefore, the focus on NO in this study is aligned with the current research trajectory that seeks to understand and harness its multifaceted role in improving plant resilience to drought, which is increasingly important in the context of global water scarcity and climate change (Seleiman et al. 2021).

The post-translational modifications (PTMs) induced by NO, such as S-nitrosylation, are essential for altering protein function and regulating stress responses, highlighting the sophisticated regulatory system of NO during stress conditions (Kour et al. 2023). S-nitrosylation, a critical PTM induced by nitric oxide (NO), plays a vital role in plant responses to drought stress (Nabi et al. 2019). This PTM involves the addition of an NO group to the thiol group of cysteine residues in proteins, leading to the formation of S-nitrosothiols (SNOs). S-nitrosylation can alter protein function, stability, and interaction with other biomolecules, thereby regulating a wide range of physiological processes (Wei et al. 2020). Additionally, S-nitrosylation influences the antioxidant defense system by modulating the activity of enzymes that scavenge ROS, thus protecting plants from oxidative damage caused by drought stress (Zhang and Liao 2022; Samanta et al. 2023). Numerous proteins are targeted by both NO and H_2_S to stimulate PTMs, including S-nitrosylation and persulfidation resulting from oxido-nitrosative processes (Wang et al. 2021). Notably, the interaction between NO and ROS contributes to dynamic oxido-nitrosative processes. This intricate network has been reported to involve the –CO group in protein carbonylation and regulatory enzymes such as GSNOR and NOX (Valderrama et al. 2019). The significance of S-nitrosylation in this research article lies in its potential to enhance the understanding of NO-mediated signaling pathways and their role in improving plant tolerance to drought stress.

While NO’s role as a signaling molecule in enhancing plant tolerance to various abiotic stresses has been well-documented (Gupta and Seth 2020, 2023; Mariyam et al. 2023; Prajapati et al. 2023), its specific effect on oxidative and nitrosative processes under drought stress in tomato plants remains less explored. Previous studies have highlighted the importance of NO in drought stress signaling and tolerance in plants, emphasizing its role in activating ROS scavenging enzymes and regulating antioxidative systems (Chavoushi et al. 2019; Jafari and Shahsavar 2022). However, the intricate mechanisms by which NO modulates oxidative and nitrosative processes, particularly in tomato plants under drought stress, have not been thoroughly investigated. Building on this, our study explores the cross-talk between NO and hydrogen sulfide (H_2_S), mediated by l-cysteine desulfhydrase (L-DES) activity, as a novel area of investigation. This cross-talk is proposed as a crucial mechanism impacting plant responses to drought stress. The interaction between NO and H_2_S may lead to modifications in protein function and signaling pathways, which are essential for the plant’s adaptation to water scarcity. The role of L-DES in mediating the production of H_2_S from l-cysteine has been recognized as a significant factor in plant stress physiology (Shen et al. 2019; Muñoz-Vargas et al. 2022). Furthermore, the interplay between NO and H_2_S has been shown to involve various post-translational modifications, including S-nitrosylation and persulfidation, which affect protein activity and cellular signaling under stress conditions (Aroca et al. 2018; Singh et al. 2022). These modifications are crucial for the regulation of stomatal closure, antioxidant defense, and hormonal signaling, which collectively contribute to the plant’s drought tolerance (Lau et al. 2021; Yasir et al. 2023).

## Materials and methods

### Cultivation practices and treatments

Tomato (*Lycopersicum esculentum*) cultivar “SC 2121” were grown in the greenhouse. The seeds of the tomato cultivar ‘SC 2121’ were sourced from Connect Anadolia seed company, based in Izmir, Turkey. This variety was selected for its early growth, suitability for field cultivation and table consumption, and desirable characteristics such as being round, red, and thin-shelled. After being surface sterilised with 1% sodium hypochlorite solution (v/v), tomato seeds were placed in a dish composed of cleaned sand and given water each day to germinate. The greenhouse maintained optimal environmental settings for the tomato, with nighttime temperatures within the range of 12–15 °C and daytime temperatures ranging between 20 and 30 °C. Throughout the experiment, the light period consistently lasted about 11 h, and the relative humidity was maintained between 60 and 70%. One week after germination and before stress treatment, the control (C; no PEG application) and DS seedlings were subjected to a pretreatment with 0.2 mM sodium nitroprusside (SNP), which is an NO donor. In addition, 0.1 mM cPTIO (NO scavenger) was also supplied through 10% Hoagland nutrient solution for 24 h. After 24 h of pretreatment with NO/cPTIO, to induce drought stress conditions mimicking drought stress in relevant groups of seedlings, DS seedlings were treated with a nutrient solution diluted by half, supplemented with 12% PEG for 48 h. This osmotic stress limits plants' ability to absorb water, modifying water deficiency stress. Then both control (C, no PEG application) and DS-plants were further grown in half-strength NS for 10 days.

Our study employed a randomized full-block design with three replications to assess the effects of drought stress on plant growth. Each replication consisted of three seedlings planted in individual 5 L containers. These containers were filled with a half-strength Hoagland nutrient solution to ensure consistent nutrient availability.

**Fig. 1:**
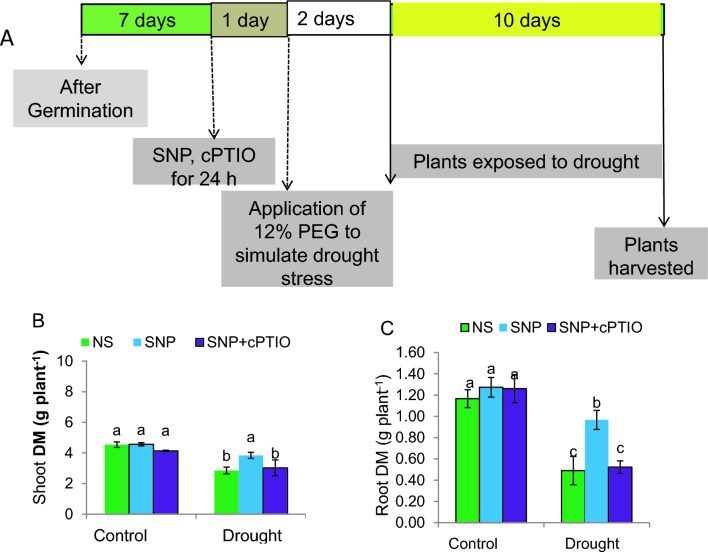
**A** Experimental protocol overview: One week post-germination, control (C) and drought-stressed (DS) seedlings received pretreatment with 0.2 mM sodium nitroprusside (SNP) and 0.1 mM cPTIO (NO scavenger) for 24 h. Subsequently, DS seedlings were exposed to 12% PEG for 48 h to induce drought stress, while control seedlings remained untreated. Both groups were then maintained in half-strength nutrient solution for 10 days. **B, C** Shoot (**B**) and root (**C**) dry matter (DM) measured in phenotyping experiments. NS stays for “not sprayed” (e.g.—the mock control; plants sprayed with distilled water). SNP- plants pretreated with 0.2 mM of sodium nitroprusside; cPTIO – plants pretreated with a scavenger of nitric oxide. Data is mean ± S.E. (*n* = 6–8). Bars assigned different low-case letters indicate a statistically significant difference at *P* ≤ *0.05*

The details of these treatments are summarized in Fig. 1A, which is included to enhance comprehension of the experimental design. The treatments were as follows:

1. Control Group: Seedlings were treated with a mock solution to serve as a baseline for comparison.
2. SNP Treatment Group: Seedlings were subjected to the Control group and treated with sodium nitroprusside (SNP), a known donor of NO.
3. SNP + cPTIO Treatment Group: Seedlings experienced Control group and received a combination of SNP and cPTIO, scavenger of NO.
4. Drought Stress Group: Seedlings underwent drought conditions and were also treated with the mock solution.
5. SNP Treatment Group: Seedlings were subjected to drought stress and treated with sodium nitroprusside (SNP), a known donor of NO.
6. SNP + cPTIO Treatment Group: Seedlings experienced drought stress and received a combination of SNP and cPTIO, scavenger of NO.

Each treatment was replicated three times, and within each replicate, there were three pots, each containing three plants. This resulted in a total of nine plants per treatment across all replicates. The pH level of the NS was adjusted to 5.5 using a small amount of solution of KOH (0.01 M).

At the end of the 10-day treatment period, three plants selected, one from each replicate, for each treatment were harvested to measure their dry weight. The plants were allowed to air-dry before undergoing a 10-min exposure to 105 °C in an oven before being maintained at 72 °C for an extra 72 h to achieve a constant dry weight. The two additional plants in each replicate (resulting in six plants per treatment) were uprooted to quantify the parameters listed below:

### Measurement of chlorophylls and chlorophyll fluorescence

For chlorophyll measurement in plant samples, we employed the Strain and Svec (1966) method. This technique entails pigment extraction using a solvent, followed by absorbance measurement with a spectrophotometer. Homogenization of 1 g of leaf tissue was carried out in 5 mL of 90% acetone. The extracts were maintained in sealed tubes after filtration. Concerning chlorophyll a and chlorophyll b, corresponding absorbance measurements were recorded at 663.5 nm and 645 nm using a spectrophotometer, and chlorophyll concentrations were determined using the formula outlined by Lichtenthaler and Wellburn (1983).

The leaves of tomato underwent standard light and dark periods, a widely used technique for assessing levels of chlorophyll fluorescence. Our analysis benefited from the precise and reliable data provided by the MINI-PAM-II Photosynthesis Yield Analyzer (Model No. PYAA0664)**,** manufactured by Heinz Walz GmbH, Germany**,** which features a 735 nm LED and PAR sensor among its advanced functionalities. Before acquiring the fluorescence measurements, the leaves were placed in the absence of light for thirty minutes. This darkness period was crucial for determining the maximum quantum efficiency of PSII, denoted as *Fv/Fm*. This specific value holds significance in the analysis of photosynthetic activity, representing the efficiency of energy conversion into chemical energy during photosynthesis in plants.

### Quantification of leaf water potential (Ψ_I_) and determination of relative water content (RWC)

The Ψ_I_ of the third uppermost leaf was measured utilizing a pressure chamber, adhering to standard protocols and employing a Ψ_I_ measuring device (USA-made PMS model 600). The process comprises the step of fixing a leaf from the plant onto a chamber and subsequently sealing it securely. The pressure is systematically raised until the first appearance of water droplets at the tip of the petiole. The corresponding pressure level recorded by the pressure chamber at this juncture is recorded as the Ψ_I_ of the leaf.

The RWC was computed using the Barrs and Weatherley (1962) technique. Initially, fresh leaves from the corresponding part of the plant were swiftly weighed to determine their FW (fresh weight). Subsequently, these leaves underwent a one-day incubation in vials filled with distilled water. Once the leaves were removed from the vials, the removal of any surface water was accomplished by gently wiping it off employing a paper towel to absorb excess moisture, and the leaf TW (turgid weight) was determined. The subsequent procedure included dehydrating the samples consistently in an oven set at 65 °C to determine the leaf DW (dry weight). The equation used for estimating the Relative Water Content (RWC) as per Barrs and Weatherley (1962) is as follows:

$${\text{RWC}}(\% )\, = \,\left( {{\text{FM}}{-}{\text{DM}}} \right) \, / \, \left( {{\text{TM}}{-}{\text{DM}}} \right)\, \times \,{1}00$$

### Processing and analysis of thermal images

Infrared imaging through a FLIR T540 thermal camera was utilized to monitor the plant canopy temperatures at their warmest during the daytime. The thermal camera, positioned on a tripod and calibrated for emissivity, recorded images of the complete canopy. Utilizing FLIR Tools software, the images underwent processing to quantify temperatures, and subsequently, the average temperature readings were computed. In order to guarantee a sample representative of the canopy, in total, eight readings were recorded, with two readings collected from every facets of the plant.

### Determination of free proline content

To determine the leaf-free proline concentration, we followed the experimental approach detailed according to Bates et al. (1973) methodology. The procedure included extracting a half g leaf sample with a 3% of 10 mL sulfosalicylic acid solution. Next, a 2 mL portion of the filtrate was combined with 2 mL each of 2.5% acid-ninhydrin (w/v) and 60% glacial acetic acid (v/v) solutions. The resulting reaction solution was then subjected to heating for an hour in a water bath with a temperature set to 100 °C, following that, it was permitted to cool down. The cooled solution was supplemented with 4 mL toluene, and the mixture’s absorbance was subsequently measured at 520 nm. Subsequently, the absorbance data was employed to calculate free proline concentration. This quantification was achieved by referencing a standard graph constructed with known quantities of proline added.

### Hydrogen peroxide (H_2_O_2_) content

The H_2_O_2_ concentration was calculated using the Sergiev et al. (1997) protocol. Centrifugation of the fresh samples was performed at 12,000×g at 4 °C for 15 min after being homogenised in TCA (0.1%). A 1 M potassium iodide, which is produced in phosphate buffer, was used to treat the supernatant. The mixture's absorbance was noted at 390 nm. This reading was then employed to compute the H_2_O_2_ concentration, referring to a standard graph constructed using established H_2_O_2_ concentration.

### The extraction of protein

To measure the protein concentration, the Bradford (1976) procedure was employed. After being powdered using liquid nitrogen, the samples underwent homogenization in a 100 mM Tris–HCl extraction buffer (pH 8.0) prepared in a solution of 10% glycerol (v/v), 5 mM DTT, 1 mM EDTA, and 0.02% Triton X-100 (v/v). This extraction buffer helps to maintain the integrity of the proteins and prevent their denaturation during the extraction process. Subsequently, the supernatant, representing the liquid fraction, was carefully extracted after centrifugating at 17,000*g* for 20 min at 4 °C, effectively separating it from the cellular remnants. Following centrifugation, the supernatant was acquired for evaluating the protein concentration, along with other assessments of enzyme activity, protein carbonyl groups (–CO). and thiol groups (–SH). To determine the protein concentration, the Bradford (1976) method was used, which relies on the interaction with Coomassie Brilliant Blue dye to the protein molecules, leading to a shift in the dye’s absorbance spectrum. The sample optical density was recorded through absorbance measurements at 595 nm, and the protein concentration was computed using a calibration plot generated using known bovine serum albümin concentrations.

### The quantification of protein –CO and –SH groups

For protein –CO content measurement in the extracted proteins, a solution comprising 6 M guanidine and 10 mM DNPH was formulated in 2 N HCl as solvent. The protein extract was subsequently subjected to incubation in this solution. Spectrophotometric analysis at a wavelength of 480 nm was employed for calculating the –CO contents, following the method detailed in the study by Levine et al. (1994). To determine the levels of –SH in the protein extracts, the increment in absorbance at 412 nm due to DTNB reduction in 0.05 M Tris–HCl (pH 8) was measured. This procedure was carried out following the approach detailed in the study by Ellman (1959).

### Analysis of enzyme activity

The SOD activity was determined by the degree to which it inhibited the photoreduction of NBT (Van Rossum et al. 1997). The quantity of needed enzyme to achieve a 50% reduction in cytochrome c levels was quantified as SOD activity. The technique introduced by Fielding and Hall (1978) was used to determine the GPX activity. Its activity was evaluated by measuring absorbance increase at 470 nm due to the polymerization of guaacol into tetragacol when H_2_O_2_ is present. Spectrophotometer was used to measure the GSNOR activity at 25 °C by monitoring the NADH oxidation at 340 nm (Barroso et al. 2006). The initiation of the enzymatic process commenced with the addition of GSNO into the reaction mixture. Enzyme activity measurement relied on the utilization of NADH, enabling the measurement of its activity through the rate of either NADH consumption or NADH production. The assessment of NOX activity was conducted according to the principle of NADPH oxidation leading to the formation of NADP + , with absorbance at 340 nm. The NOX activity was assessed through tracking the decline in absorbance at 340 nm, as outlined by Ishida et al. (1987).

### Measurement of NO and S-nitrosothiol (SNO)

Adhering to the procedure outlined by Kaur et al. (2015), the Griess reagent was employed to assess the NO concentration in a solution of 5% phosphoric acid. Fresh samples were homogenized and then underwent centrifugation at 12,000*g* at 4 °C for 10 min. To ascertain the NO concentration, the Griess reagent-supernatant mixture underwent a 10-min incubation at room temperature, shielded from light. During this incubation period, the Griess reagent was permitted to undergo a reaction with NO, producing a colored compound measurable with spectrophotometer. Post-incubation, the absorbance of the resulting solution was recorded at 540 nm. To quantify NO concentration, a calibration curve was generated using NaNO_2_.

The extraction of SNO was carried out following the methodology detailed by Gow et al. (2007). Leaf material was extracted using 50 mM phosphate buffer (pH 7.2) with the addition of 80 mM S-methyl methanethiosulfonate. Following centrifugation at 20,000*g* for 20 min at 4 °C, the resultant solutions were collected and mixed with cold acetone. Subsequent centrifugation and a 60-min incubation at 24 °C were performed before re-suspending the recovered pellets in the extraction buffer. The measurement of SNO was then carried out following the procedure outlined by Saville (1958). For a duration of 20 min, a 0.3 mL protein extract sample was combined with a solution consisting of 3.4% sulfanilamide (prepared in 0.4 M HCl) and 0.1% *N*-(1-naphthyl) ethylenediamine. This solution selectively blocked the reaction of specific –SH groups, facilitating the measurement of distinct subsets of –SH groups. Following a 20-min incubation at ambient temperature, the absorbance reading at 540 nm was recorded.

### Measurement of AsA and GSH contents

For the analysis of AsA and GSH, a half g fresh leaf sample was utilized. The extraction process involved a 3 mL cold solution consisted of 5% meta-phosphoric acid and 1 M EDTA. After centrifugation, measurements for AsA and GSH were performed following the procedure detailed by Huang et al. (2005). The measurement of reduced glutathione (GSH) and glutathione disulfide (GSSG) levels utilized a procedure detailed by Yu et al. (2003). After neutralizing the sample solution with a 500 mM K-phosphate buffer, the GSH level was determined at 412 nm by monitoring changes in optical density values resulting from the reduction of 5,5'-dithiobis (2-nitrobenzoic acid). GSSG concentration was calculated by subtracting the 2-vinyl pyridine content from the GSH content.

### Statistical analysis

A one-way ANOVA was conducted on the data to assess potential significant differences among various parameter settings. Mean values and standard errors were calculated and are visually illustrated in the figures. The determination of statistical significance for observed differences adhered to a significance level of 0.05%.

## Results

### SNP promotes plant growth under DS

Exposure to PEG-induced DS resulted in a significant reduction (*P* ≤ *0.05*) of shoot and root dry mass of plants by 37% and 58%, respectively, in comparison to untreated control plants. However, application of 0.2 mM SNP, a donor of NO, significantly improved plant growth-related variables by 28% and 98% for shoot and root dry mass, respectively, when compared to plants experiencing DS alone (Fig. 1B, C), but the SNP-related results on improved plant growth parameters remained lower than those in C-plants.

To assess whether nitric oxide (NO) produced by SNP (sodium nitroprusside) had a mitigating effect on drought stress, we combined SNP treatment with the NO scavenger, cPTIO, in settings with normal conditions and drought stress. The addition of 0.1 mM cPTIO along with SNP treatment resulted in the loss of the positive effects of SNP on plant triats in drought-stressed plants. No changes in these characteristics were observed in control plants due to different treatments.

### SNP promotes photosynthesis-related parameters under DS

**Fig. 2:**
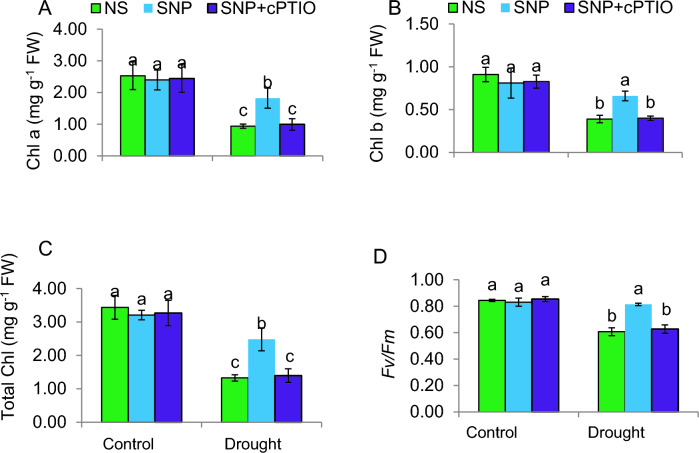
Leaf photosynthetic characteristics of pepper plants under control and PEG-stimulated drought conditions. **A, B** Chlorophyll *a* and *b* content; **C** Total chlorophyll content; **D** Chlorophyll fluorescence *Fv*/*Fm* ratio. NS = mock control (plants sprayed with distilled water). SNP- plants pretreated with 0.2 mM of sodium nitroprusside; cPTIO – plants pretreated with a scavenger of nitric oxide. Data is mean ± S.E. (*n* = 6–8). Bars assigned different low-case letters indicate a statistically significant difference at *P* ≤ *0.05*

The levels of chlorophyll a and b, total chlorophyll, and photosystem II efficiency (*Fv/Fm*) were significantly reduced (*P* ≤ *0.05*) in the DS tomato seedlings. Specifically, in comparison of C plants, the concentrations of chlorophyll a and b decreased by 54% and 30%, respectively. Furthermore, the total chlorophyll concentration and *Fv/Fm* in DS plants were decreased by 6142% and 27%, respectively. However, SNP treatment led to significant elevations in these photosynthesis-related parameters, with 72%, 39%, 56%, and 34% in chlorophyll a and b, total chlorophyll, and *Fv/Fm*, respectively (Fig. 2A–D), compared to plants exposed to DS. The administration of cPTIO reversed the positive impacts of SNP on these traits under drought conditions by scavenging NO, thus demonstrating that SNP increases the levels of NO, acting as a donor of NO to enhance these physiological responses in plants. Under control conditions, plants did not show any differences in these traits resulting from the various treatments.

### Exogenous SNP treatment enhances proline content, transpirational cooling and improves leaf water relations

**Fig. 3:**
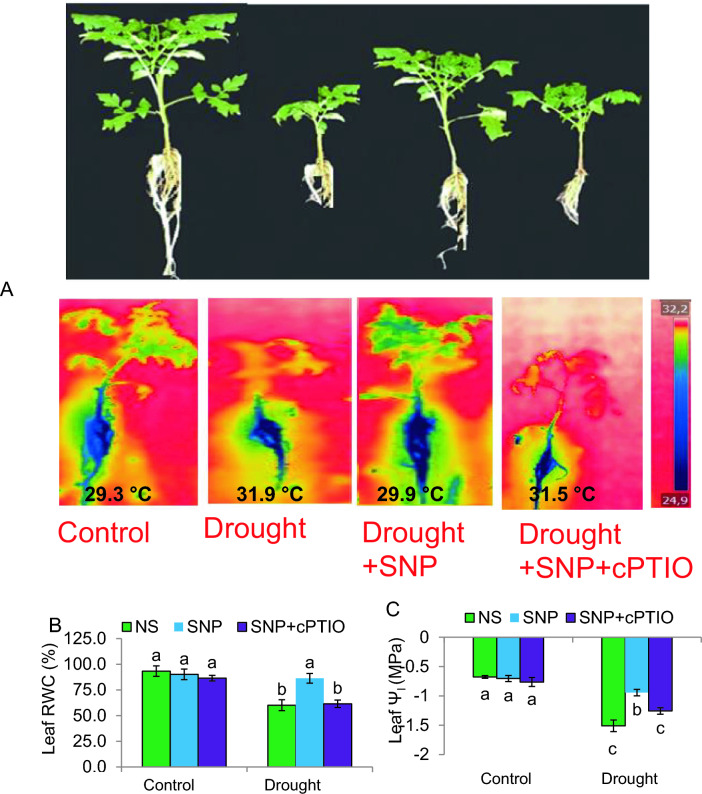
The water relations of tomato plants under PEG-stimulated drought conditions. **A** Plant phenotype and leaf temperature. **B** Leaf relative water content (RWC). **C** Leaf water potential (Ψ_l_). NS = mock control (plants sprayed with distilled water). SNP- plants pretreated with 0.2 mM of sodium nitroprusside; cPTIO – plants pretreated with a scavenger of nitric oxide. Data is mean ± S.E. (*n* = 6–8). Bars assigned different low-case letters indicate a statistically significant difference at *P* ≤ *0.05*

Plants affected by drought exhibited wilting signs (Fig. 3A), which were not observed in plants treated with SNP. The positive effect of SNP on alleviating wilting symptoms of DS was nullified by pretreatment with cPTIO. An infrared image was utilized for measuring the canopy temperatures of tomatoes that were subjected to various treatments. Compared to the control treatment, drought significantly raised leaf temperatures from 29.3 to 31.9 °C. However, SNP application significantly lowered the canopy temperature of DS plants to 29.9 °C (Fig. 3A), indicating an improvement in transpiration-induced cooling. However, the positive effect of NaHS in reducing canopy temperature was nullified by cPTIO, which is a scavenger of NO. This finding suggests that the lower canopy temperature observed with SNP treatment is possibly attributed to the NO application, which enhances the plants' ability to tolerate DS.

**Fig. 4:**
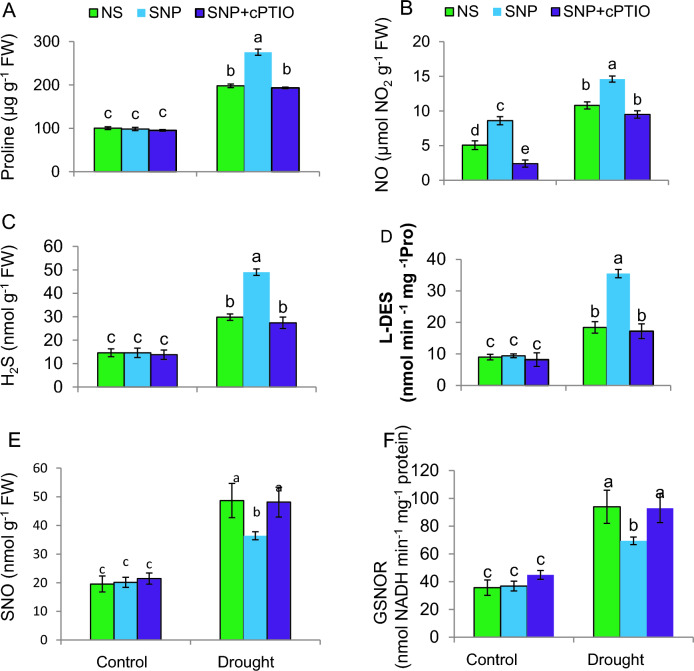
Biochemical modifications in tomato plant under PEG-stimulated drought conditions. **A** Proline content; **B** Nitric oxide content (NO); **C** Hydrogen sulphide (H_2_S); **D** l-cysteine desulfhydrase (L-DES); **E** *S*-nitrosothiol (SNO) on fresh weight (FW) basis; **F** *S*-nitrosoglutathione reductase (GSNOR). NS = mock control (plants sprayed with distilled water). SNP- plants pretreated with 0.2 mM of sodium nitroprusside; cPTIO – plants pretreated with a scavenger of nitric oxide. Data is mean ± S.E. (*n* = 6–8). Bars assigned different low-case letters indicate a statistically significant difference at *P* ≤ *0.05*

DS decreased tomato leaf RWC and Ψ_I_ to 35% and 125%, respectively, but increased the proline content of tomato plants to 120% (Figs. 3B–C, 4A). SNP, a NO donor, was employed to test whether exogenous NO is capable of altering how tomato seedlings respond to water deficiencies. When compared to drought-stressed plants alone, SNP elevated leaf RWC, Ψ_I_ and proline by 41%, 37% and 39%, respectively. None of the treatments had an effect on the C-plants. Plants supplied with cPTIO, an NO scavenger, together with SNP abolished the benefits of SNP on those traits. SNP produces NO and increases leaf water relation and proline while scavenging NO renders SNP ineffectual in increasing leaf water status.

### SNP modulates H_2_S, L-DES and nitrosative potential: crosstalk between H_2_S and NO signaling

The observed increases in endogenous NO and H_2_S levels, along with L-DES activity in drought-stressed plants treated with SNP. This suggests a synergistic action where NO may enhance H_2_S synthesis, contributing to the overall stress response. The elevation of endogenous NO and H_2_S, both signaling molecules, indicates their role in activating stress-responsive pathways. (Fig. 4B–D). Conversely, the reduction in SNO content and GSNOR activities upon SNP treatment alone suggests that NO signaling is tightly regulated with S-nitrosylation during stress responses. Under DS, the combined treatment of cPTIO and SNP increased the SNO content and GSNOR activity while decreasing the NO and H_2_S generation, as well as L-DES activity (Fig. 4E, F), highlighting the complexity of NO signaling and its interaction with H2S. This indicates that the presence of cPTIO alters the balance of NO and H2S, potentially through the modulation of S-nitrosylation processes. In contrast, under control conditions, the SNP application elevated the NO content, while the combined treatment of cPTIO and SNP decreased it. The H_2_S content, L-DES activity, SNO concentration, and GSNOR activity in control plants were not affected by any treatment, indicating the specificity of the responses observed under drought stress conditions and highlighting the complex regulatory mechanisms governing NO and H_2_S signaling pathways in tomato plants.

This intricate crosstalk between NO and H_2_S is crucial for the regulation of plant physiological processes, particularly under stress conditions. The mutual influence on each other’s production and the downstream signaling pathways they impact are central to the plant’s ability to adapt and survive under adverse conditions.

### SNP modulates antioxidant enzymes and oxidative stress markers

**Fig. 5:**
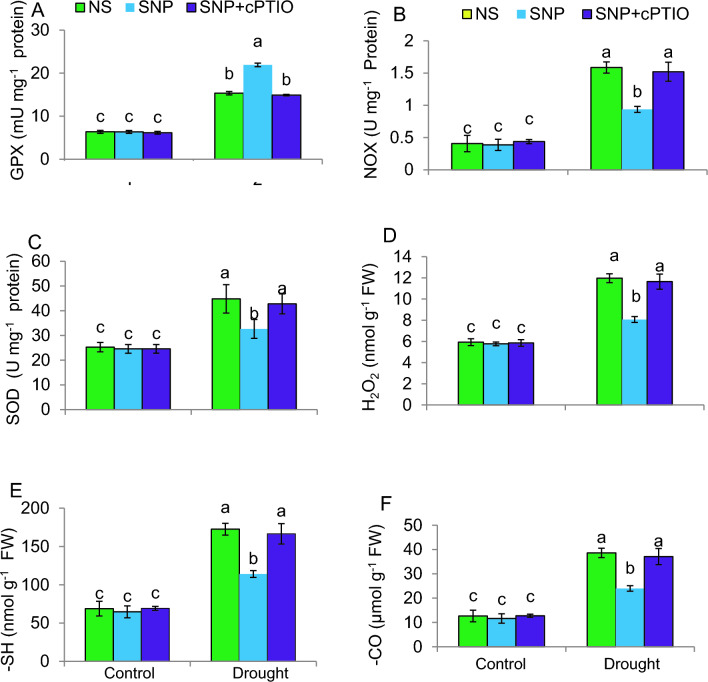
Activities of key antioxidant enzymes and redox characteristics of tomato plants under control or PEG-stimulated drought conditions. **A** Glutathione peroxidase (GPX); **B** NADPH oxidase (NOX); **C** Superoxide dismutase (SOD); **D** Hydrogen peroxide (H_2_O_2_) on fresh weight (FW) basis; **E** Thiol (–SH); **F** Carbonyll (–CO). i NS = mock control (plants sprayed with distilled water). SNP- plants pretreated with 0.2 mM of sodium nitroprusside; cPTIO – plants pretreated with a scavenger of nitric oxide. Data is mean ± S.E. (*n* = 6–8). Bars assigned different low-case letters indicate a statistically significant difference at *P* ≤ *0.05*

The GPX, NOX, and SOD activities and H_2_O_2_ levels exhibited a significant elevation in response to DS (Fig. 5A–D). In contrast to plants exposed to drought alone, treatment with SNP significantly (*P* ≤ 0.05) decreased NOX, SOD, and H_2_O_2_ levels by 41%, 27%, and 33%, respectively, but dramatically increased GPX activity by 49%. No notable changes in these parameters were noted in the control groups due to the different treatments. Scavenging NO buildup with cPTIO pre-treatment counteracted the effects of SNP on these traits.

The levels of carbonyl (–CO) and thiols (–SH) were considerably increased by drought stress, but treatment with SNP reduced these levels (Fig. 5E, F). The control plants showed no significant changes in these traits when subjected to the different treatments. However, pre-treatment with cPTIO reversed the effects of SNP on these traits. The control plant variables remained unchanged following the different treatments.

### SNP improves non-enzymatic antioxidants, and reduces oxidized glutathione under drought

**Fig. 6:**
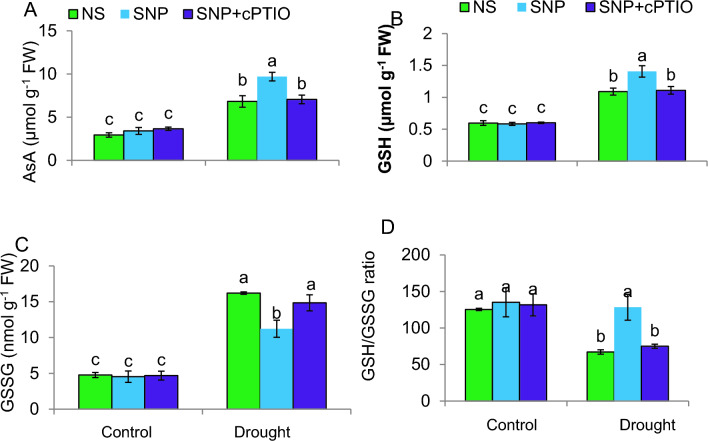
Non-enzymatic antioxidants and oxidized glutathione of tomato plants under control or PEG-stimulated drought conditions. **A** Ascorbate (AsA); **B** Reduced glutathione (GSH); **C** Oxidized glutathione (GSSG) on fresh weight (FW) basis; **D** GSH/GSSG ratio. NS = mock control (plants sprayed with distilled water). SNP- plants pretreated with 0.2 mM of sodium nitroprusside; cPTIO– plants pretreated with a scavenger of nitric oxide. Data is mean ± S.E. (*n* = 6–8). Bars assigned different low-case letters indicate a statistically significant difference at *P* ≤ *0.05*

Drought stress dramatically boosted leaf AsA and GSH concentrations, with a 134% increase in AsA and an 83% elevation in GSH compared to C plants (Fig. 6A, B). Additionally, supplementation with SNP further elevated AsA and GSH levels by 42% and 28%, respectively, compared to the levels observed in plants exposed to drought conditions. This shows that SNP may protect plants against oxidative stress due to DS.

Drought stress increased the quantity of oxidized glutathione (GSSG) by 226% when compared to C plants (*P* ≤ *0.05*) (Fig. 6C). However, the use of SNP led to a notable reduction in GSSG content when compared to plants experiencing drought stress alone. Drought stress decreased the GSH/GSSG ratio in plants (Fig. 6D), but SNP application increased the ratio, indicating a potential positive effect on redox balance. The cPTIO fully reversed the increase in AsA and GSH caused by SNP under DS. This suggests that SNP provides NO, which in turn enhances AsA and GSH concentrations while reducing the concentration of GSSG. These results suggest that the defensive impacts of SNP on plants under drought stress may be attributed to this potential mechanism. Other treatments, on the other hand, had no effect on these compounds in plants that were not under stress, indicating that the observed alterations in these compounds were specific to the plant response to DS and the application of the treatments.

## Discussion

### SNP improves critical growth traits under DS

Drought stress has the potential to disturb the water balance in plants, leading to compromised physiological processes such as growth, leaf water status, and oxidative stress (Hosseini et al. 2019; Saja-Garbarz et al. 2021; Wahab et al. 2022). In response to DS, plants employ adaptive strategies, including the production of osmolytes like proline, to facilitate enhanced water uptake from the root zone, as proposed by Hosseinifard et al. (2022). In the face of DS, our study uncovered a notable decline in both leaf RWC and water potential (Ψ_l_) among the affected plants. In response to drought conditions, there was a notable increase in proline content, indicating the plant’s effort to sustain its water status (Pandey and Shukla 2015; Hanif et al. 2021). However, the introduction of SNP resulted in enhanced leaf Ψ_l_, RWC, and proline content of drought-stressed plants. This suggests that SNP has the potential to ameliorate water content in plants experiencing drought stress. Consistent with our findings, prior studies have indicated that SNP-induced alterations in osmoregulation in marjoram herb contributed to heightened proline content and improved water potential (Farouk and Al-Ghamdi 2021). One potential explanation for the observed improvement in plant growth following treatment with SNP under drought stress, as proposed by Faraji and Sepehri (2020) in wheat plants, is that SNP enhances proline content, this improves the plant water status under DS.

To clarify the function of NO in the increased DS tolerance of tomato treated with SNP, the plants received treatment with a NO scavenger, specifically cPTIO. The results showed that the cPTIO application inverted the beneficial impacts of SNP on plant growth under drought stress, indicating that NO generated by SNP is essential for the improved DS tolerance of tomato plants. The findings also show that the observed enhancement in plant growth induced by SNP during drought stress may be due to NO accumulation. Numerous studies have used cPTIO to elucidate the role of NO in boosting stress tolerance. For instance, Kataria et al. (2020) and Wei et al. (2022a) demonstrated the involvement of NO in improving the tolerance of soybean and tomato to salt stress by using cPTIO as a NO scavenger. Therefore, the use of cPTIO in this study is consistent with previous research and helps to confirm the role of NO in the observed enhancement in drought tolerance of tomato plants treated with SNP.

### SNP enhances photosynthesis-associated traits under drought

In the presence of drought stress, crucial parameters for photosynthesis in tomato plants, such as Fv/Fm and chlorophyll content, experienced a negative impact. This aligns with prior studies that have consistently reported comparable reductions in chlorophyll content and Fv/Fm in various plant species facing drought conditions (Badr and Brüggemann 2020; Larouk et al. 2021). The imposition of DS can result in elevated levels of the enzyme chlorophyllase, subsequently impeding chlorophyll production (Altuntaş et al. 2020); this phenomenon may be a contributing factor in the observed decrease in chlorophyll levels under DS.

However, in the current study, the SNP treatment positively influenced photosynthetic attributes while mitigating the accumulation of H_2_O_2_, a potential threat to photosynthetic machinery. These outcomes align with previous research showing the beneficial impact of SNP on photosynthetic traits in wheat plants subjected to drought (Faraji and Sepehri 2020).

Under DS, SNP application in tomato plants acted as a donor of NO, resulting in a significant rise in NO content. The application of SNP in tomato plants subjected to DS resulted in a notable elevation in NO content, which is involved in promoting plant growth and enhancing stress tolerance. This was substantiated by the enhanced *Fv/Fm* and chlorophyll content, indicating the beneficial influence of NO on photosynthetic efficiency and overall plant health. To verify the involvement of NO in these beneficial effects, cPTIO, a NO scavenger, was administered alongside SNP. The findings showed that the cPTIO application inverted the beneficial effects of SNP on increases in *Fv/Fm* and chlorophyll content, providing more proof regarding NO’s participation in the action mechanism of SNP in enhancing plant growth and tolerance to DS.

### SNP controls nitrosative stress in DS conditions

The tomato plants that were fed with SNP showed significant increases in NO and H_2_S concentrations. This demonstrates how NO activates H_2_S as a downstream signal mediator in declining oxidative stress caused by drought. This boosts the antioxidant defense system, hence increasing the tomato plant’s tolerance to drought. Under drought, NO was observed to build up in various plants, including *Lotus japonicas* (Signorelli et al. 2013), and maize (Majeed et al. 2020). Likewise, the accumulation of H_2_S has been documented in wheat (Li et al. 2021) and tomato (Siddiqui et al. 2021) under DS. Numerous studies have indicated that DS increases ROS formation and oxidative stress (Sohag et al. 2020; Zou et al. 2021). In the face of oxidative stress, plants can naturally generate both NO and H_2_S as components of their strategy for safeguarding against stress (Zhu et al. 2018). These signaling molecules have been demonstrated to safeguard plants against oxidative stress and to promote plant survival during drought conditions (Li et al. 2016; Antoniou et al. 2020). By upregulating the levels of NO and H_2_S levels, plants can initiate their antioxidant defense system, stimulate stress-induced gene expression, and modulate stomatal closure. These mechanisms are involved in reducing the adverse impacts of oxidative stress and other environmental stressors (Bhuyan et al. 2020; Gautam et al. 2022). Through the implementation of these adaptive strategies, plants enhance their ability to withstand and recover from various environmental stressors, thereby contributing to improved overall growth and survival.

Furthermore, SNP treatment during drought induced an increase in NO buildup and H_2_S synthesis. Exogenous SNP enhances NO buildup in safflower (Chavoushi et al. 2020) and maize (Majeed et al. 2020) under water stress. In addition, in a study by Alamri et al. (2020), it was noted that exposure to hazardous amounts of chromium significantly elevated the activity of L-DES and D-cysteine desulfhydrase (D-CD), leading to an elevation in H_2_S production in tomato plants. Similarly, in our investigation, the SNP application also enhanced the H_2_S content by enhancing the activity of L-DES. Both findings indicate that plants H_2_S production can be stimulated by various stressors, including Cr(VI) toxicity and DS, and that this production can be further elevated due to SNP application. These two signalling molecules have been shown to have overlapping and sometimes antagonistic functions in facilitating plant adaptation to abiotic stress (Kharbech et al. 2020b).

Crucially, it should be acknowledged that NO and H_2_S, along with their derivatives, have the capability to interact and modulate the signaling pathways of one another. This underscores the possibility of inter-molecular crosstalk and Its possible repercussions on downstream signaling processes (Singh et al. 2020). Numerous pertinent target proteins associated with these signaling molecules are susceptible to PTMs, including S-nitrosylation and persulfidation. These modifications alter the functioning of proteins, introducing a layer of complexity to cellular processes influenced by NO and H_2_S. (Corpas et al. 2021; Corpas and Palma 2023). The interplay between H_2_S and ROS/RNS in DS conditions holds considerable impact on plant growth and ability to thrive. H_2_S has demonstrated the ability to eliminate ROS/RNS, mitigating their detrimental impacts on plant cells (de Bont et al. 2022). A comprehensive understanding of the crosstalk between these molecular signals and their influence on PTMs can offer valuable perspectives on the intricate regulatory networks modulating plant stress responses.

S-nitrosylation is a prevalent cellular communication process involving reversible PTMs (Shi and Qiu 2020). Under drought conditions, there was a notable rise in the cellular SNO concentration in tomato, suggesting the presence of S-nitrosylation on R-SH, specifically targeting protein thiols. This finding aligns with observations made by Wang et al. (2022) and Wei et al. (2022b) in tomato plants subjected to salinity stress. However, SNP resulted in a decrease in SNO levels and an increase in NO concentrations in drought-exposed leaves, corroborating findings noted in tomato plants subjected to salt stress (Wang et al. 2022; Wei et al. 2022b). This complex interaction between SNO and NO signaling pathways in governing plant responses to DS indicates a nuanced regulatory mechanism. Various PTMs are involved in facilitating NO’s physiological functions, with protein S-nitrosylation, specifically the formation of an SNO through covalent bonding to the cysteine residue's reactive thiol group within a protein, being a prominent PTM (Wei et al. 2022b).

An important observation is that the NO generated through SNP application has the capacity to form covalent bonding between NO-derived species and other molecules, such as SNOs, influencing diverse plant signal transduction pathways and cellular activities (Sun et al. 2021). SNOs, originating from NO, tend to build up in plants during DS conditions, related to raised levels of oxidative and nitrosative damage (Corpas 2017). However, studies have shown that SNP can decrease SNO content in stressed-plants. For instance, Khan et al. (2019) reported that soybean plants under flooding stress and received SNP treatment showed a decline in SNO level. Furthermore, Kharbech et al. (2020a) reported a decrease in SNO level in maize plants that received SNP treatment under chromium stress. The decrease in SNO levels noted in plants that received SNP treatment under DS conditions might be ascribed to the interaction between H_2_S and SNOs. H_2_S has the ability to react with SNOs, forming substances, such as thionitrous acid, capable of decomposing into elemental sulfur and NO (Yuan et al. 2015; Benchoam et al. 2019). This reaction can lead to the transformation of SNOs into NO, consequently reducing the concentration of SNOs in the plant.

Drought stress induced an elevation in the GSNOR activity, an enzyme responsible for degrading SNOs and regulating their concentrations in plant cells. Similar observations have been documented in poplar plants exposed to chilling (Cheng et al. 2015) and heat stress (Cheng et al. 2018). The homeostasis of NO and SNOs is remarkably governed by GSNOR, which is elaborated in controlling actions mediated by reactive nitrogen species (RNS) (Badiani et al. 2022). In this study, DS-induced GSNOR activity resulted in an elevated level of NO. GSNOR believed to be the primary enzyme that regulates NO levels within cells, catalyzes the reduction of S-nitrosoglutathione (GSNO), a primary reservoir of NO, in an NADH-dependent process (Treffon and Vierling 2022).

Furthermore, the increased NO content triggered by DS was in tandem with heightened activity of L-DES and increased H_2_S production. The interaction between NO and H_2_S can result in the generation of new nitrosothiols, influencing protein activity and contributing to enhanced stress resilience (Singh et al. 2020). Conversely, elevating NO concentration due to SNP treatment can suppress the GSNOR activity, an enzyme facilitating the decomposition of S-nitrosoglutathione (GSNO) into NO and GSH (Tichá et al. 2016), resulting in NO accumulation. Additionally, SNP application increases both NO and H_2_S levels. In wheat under osmotic stress, exogenously applied NO was observed to enhance H_2_S production (Khan et al. 2017). These findings imply a complex interplay between NO and H_2_S signaling molecules in modulating plant responses to drought. Another study by Da-Silva et al. (2018) investigated the interplay between NO and H_2_S signaling pathways in regulating salinity tolerance in tomato.

### SNP lowers H_2_O_2_ through modulating ROS-scavenging enzymes and protein –CO and –SH under DS

Drought stress triggers ROS accumulation in the cell of plants, disrupting the mitochondrial electron transport machinery (Yang et al. 2021). This ROS generation process has been associated with perturbed photosynthetic performance in plants experiencing stress. This results in a surplus accumulation of energy that cannot be efficiently used for plant development and growth (Hasanuzzaman et al. 2020). Multiple studies have demonstrated that NOX serves as a principal origin of ROS generation during water stress within a spectrum of plant species, such as rice (Jing et al. 2021) and maize (Demiralay et al. 2022). It is activated by various signaling pathways in response to DS, including abscisic acid (ABA) signaling, calcium (Ca^2+^) signaling, and MAPK signaling results in ROS generation in the vicinity of the stress stimulus (Verma et al. 2019; Takata et al. 2020). The current study revealed that DS caused an elevated H_2_O_2_ in tomatoes. However, this augmentation could be moderated by SNP treatment. Furthermore, NOX activity was observed to elevate in response to water stress, a trend that was attenuated by SNP treatment. This finding was consistent with a study on cobalt-stressed wheat after SNP treatment, where NOX activity was also found to decrease (Ozfidan-Konakci et al. 2020). Additionally, a notable decline in H_2_O_2_ and NOX activity may be a possible reason for the DS tolerance observed in tomato plants following SNP treatment. In contrast to the NOX enzyme, which contributes to the harmful effects of ROS, SOD activity acts to alleviate these effects by promoting the transformation of O_2_•‾ into less harmful H_2_O_2_ and O_2_ (Islam et al. 2022). The study observed an enhancement in SOD activity due to DS, but SNP treatment did not further increase this activity. Instead, SNP markedly decreased SOD activity in tomato leaves, indicating that SNP does not promote SOD activity to counteract ROS generation in the presence of drought. Additionally, GPX enzyme activity, responsible for detoxifying H_2_O_2_ using AsA and GSH as sources of electrons (Rajput et al. 2021), increased in response to DS and was further boosted by SNP treatment in tomato leaves. This suggests that SNP treatment enhances GPX activity, potentially contributing to the plant's competence to tolerate oxidative stress induced by DS. Moreover, the recorded decline in NOX activity with SNP treatment may help reduce H_2_O_2_ accumulation, further enhancing the tomato plant's resilience to oxidative damage. Overall, these findings propose that the regulation of the activities of both NOX and GPX due to SNP may present a promising approach for bolstering plant tolerance to oxidative stress induced by drought.

The ROS generation due to stress in the cell is anticipated to result in the regulation of redox-active –SH and –CO groups on proteins (Corpas et al. 2021). This process leads to protein oxidation and the production of –CO groups. Consequently, the measurement of protein –CO groups can serve as an indicator to assess the severity of oxidative damage imposed on proteins (Heinonen et al. 2021). The protein –CO and –SH concentrations within the leaf cells of tomato seedlings were assessed following DS treatment. The SNP application led to a notable decrease in the heightened levels of –CO and –SH groups in leaf cells due to DS. This suggests that SNP exhibits the capability to alleviate the detrimental impacts of DS on protein oxidation. Under DS conditions, plants, including maize plants, have been reported to exhibit an elevation in protein –CO concentration (Warrad et al. 2020), indicating a response to increased oxidative stress during water scarcity. However, the application of SNP has been reported to mitigate the alteration of –CO groups in tomato plants under DS (Nasibi et al. 2009), consistent with the present findings. This suggests that SNP is anticipated to have a favorable effect on mitigating protein oxidation in plants under DS.

SNP's beneficial effect on reduction in ROS, carbonyl, and thiol groups, and modulating the NOX, SOD, and GPX enzymes’ activities, is dependent on the presence of NO. When cPTIO, a scavenger of NO, was used, SNP's beneficial effect on reducing oxidative stress and modulating enzyme activity was abolished. This finding supports the hypothesis that SNP, as a source of NO, improves plant resilience to DS by regulating ROS and enzyme activities. cPTIO, a compound that scavenges NO, has been reported in studies to inverse the favorable impacts of NO on plant responses to DS. For instance, in one study, the application of cPTIO eliminated the advantageous impacts of NO on antioxidant enzyme activity and ROS buildup in wheat seedlings under DS conditions (Wu et al. 2017).

### SNP upregulates non-enzymatic antioxidants under drought

Increases in the compounds that scavenge ROS and metabolites that maintain cellular redox buffering, such as GSH and AsA, represent significant adaptations that plants have evolved to cope with DS (Guo et al. 2020). One of the most crucial mechanisms for scavenging cellular H_2_O_2_ in plants involves two important metabolites, namely AsA and GSH, which have been previously reported by Kaya et al. (2020). The present findings strongly indicate that SNP treatment led to an elevation in GSH and AsA contents, contributing to the reduction of oxidative stress in drought-stressed plants. The elevation of GSH and AsA levels may have been pivotal in bolstering the antioxidant defense system, highlighting the potential of SNP to mitigate oxidative stress in plants under DS. Additionally, the decrease in GSSG levels induced by SNP is essential for ROS scavenging, maintaining the GSH redox state, and aiding in the detoxification of ROS. Hence, the lowering of GSSG levels following SNP treatment is considered a significant contributor to ROS scavenging and the alleviation of oxidative stress. Similar findings have demonstrated that SNP increased AsA and GSH levels while lowering GSSG in wheat plants under DS (Hasanuzzaman et al. 2018) and cobalt stress (Ozfidan-Konakci et al. 2020). In stress conditions, the increased ROS production can elevate GSSG levels, disrupting the GSH/GSSG ratio and possibly impairing cellular processes (Sachdev et al. 2021), as evidenced in plants under DS in our research. Failure to eliminate surplus ROS can result in oxidative damage (Mansoor et al. 2022). The current study suggests that SNP treatment leading to a higher ratio of GSH to GSSG can enhance plant resistance to DS. This finding is in agreement with earlier findings on wheat under salinity stress, which also reported a positive correlation between GSH/GSSG ratio and stress tolerance (Maslennikova et al. 2022). The increase in non-enzymatic antioxidant levels induced by SNP was reversed by cPTIO supplementation. This indicates that the increase in non-enzymatic antioxidants caused by SNP treatment is likely mediated by NO, as discussed in the review by Sharma et al. (2020). These results further support the role of NO as an important signaling molecule in plants' response to environmental stress.

## Conclusions

The study provides valuable insights into the complex cross-talk between NO and nitrosative signaling pathways, elucidating the role of sodium nitroprusside (SNP) in enhancing tomato plant tolerance to DS. Our findings demonstrate that pre-treatment with SNP effectively mitigated the adverse effects of DS on tomato plants by modulating oxidative and nitrosative processes. This included a reduction in H_2_O_2_ generation, an increase in NO content, and the stimulation of L-DES activity leading to H_2_S production. Notably, the observed decrease in the activity of GSNOR suggests a potential regulatory mechanism involving S-nitrosylation, highlighting its significance in modulating protein function and signaling pathways under stress conditions. Furthermore, SNP positively influenced non-enzymatic antioxidants such as AsA and GSH, contributing to improved plant adaptive responses to DS. These findings emphasize the importance of exploring the dynamic interplay between nitrosative and oxidative signaling pathways for a comprehensive understanding of the regulatory mechanisms governing plant responses to DS. This knowledge opens avenues for innovative strategies aimed at enhancing drought tolerance in crops.

## Future prospects

Future research endeavours could delve deeper into elucidating the specific molecular mechanisms underlying the observed interactions between NO and nitrosative signaling pathways, especially focusing on the intricate cross-talk mediated by S-nitrosylation. Additionally, exploring the downstream targets of S-nitrosylation and its impact on protein function could provide further insights into the regulatory network modulating plant responses to environmental challenges.

Furthermore, investigating the interplay between NO and H_2_S, as mediated by L-DES activity, warrants attention as it appears to represent a crucial cross-talk mechanism influencing plant adaptive responses. Unraveling the specific pathways and molecular targets influenced by this interplay could provide a more nuanced understanding of the synergistic effects between NO and H2S in enhancing drought tolerance.

Lastly, translating these findings into practical applications for crop improvement, such as the development of biofortified and stress-tolerant varieties, stands as an exciting prospect. This could involve the integration of SNP or related compounds into agricultural practices to enhance drought resilience and improve overall crop productivity in the face of changing environmental conditions.

## Data Availability

Data will be made available on request.
