# Correction to: Sodium nitroprusside modulates oxidative and nitrosative processes in *Lycopersicum esculentum* L. under drought stress

**DOI:** 10.1007/s00299-025-03529-3

**Published:** 2025-06-18

**Authors:** Cengiz Kaya, Ferhat Uğurlar, Chandra Shekhar Seth

**Affiliations:** 1 Soil Science and Plant Nutrition Department, Harran University, Şanlıurfa, 63200 Turkey; 2 Department of Botany, University of Delhi, New Delhi, Delhi 110007 India

**Correction to: Plant Cell Reports (2024) 43:152** 10.1007/s00299-024-03238-3

The authors would like to make the following correction to the above-mentioned article:

In the originally published Fig. 3A, the top panel, which displays the phenotypic appearance of tomato plants under different treatments, was inadvertently prepared using plant images from a different experimental setup. Additionally, background editing applied during figure preparation unintentionally altered the natural appearance of the plants.

To address this, the top panel has been replaced with a corrected version that accurately represents the phenotypic appearance of tomato plants corresponding to each treatment group from the correct experimental dataset. No background modifications were applied to preserve the authenticity of the plant appearance.

Please note that the bottom panel of Fig. 3A, which presents the thermal imaging of the same treatment groups, remains unchanged.

This correction has no impact on the results, data interpretation, or conclusions of the original publication.

The authors sincerely apologize for any inconvenience caused.
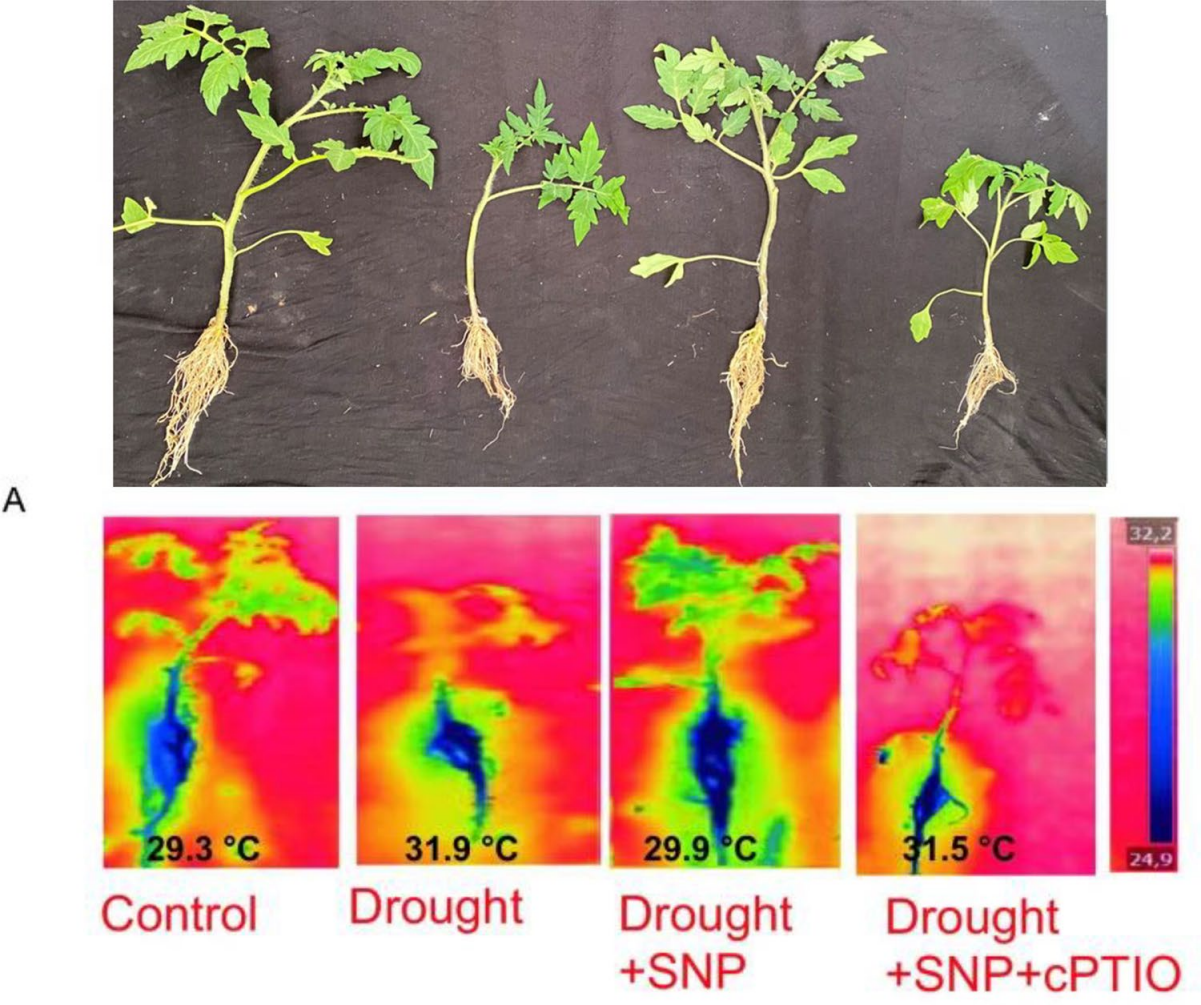


**Fig. 3A**. Phenotypic appearance of tomato plants under different treatments: control plants were well-watered and sprayed with distilled water; drought-stressed plants showed visible wilting; drought + SNP plants were pretreated with 0.2 mM sodium nitroprusside (a nitric oxide donor); and drought + SNP + cPTIO plants were treated with both SNP and cPTIO, a nitric oxide scavenger

